# Ophthalmologic emergency room visits during COVID-19 lockdown–Characterization from Haifa, Israel

**DOI:** 10.1371/journal.pone.0273033

**Published:** 2022-08-19

**Authors:** Efrat Naaman, Nitai Bar, Elie Zaher, Liran Shapira, Eytan Zeev Blumenthal

**Affiliations:** 1 Department of Ophthalmology, Rambam Health Care Campus, Haifa, Israel; 2 Department of Radiology, Rambam Health Care Campus, Haifa, Israel; 3 Ruth and Bruce Rappaport Faculty of Medicine, Technion—Israel Institute of Technology, Haifa, Israel; UNITED STATES

## Abstract

**Purpose:**

To characterize quantitative differences among ophthalmologic emergency room (OER) encounters at Rambam Health Care Campus during a 6-week complete lockdown at the peak of the first COVID-19 wave as compared to a corresponding uneventful period a year earlier.

**Methods:**

A retrospective chart analysis of all OER encounters during the lockdown and non-lockdown period was conducted. Patients were stratified into primary ophthalmological conditions (OER visits) and cases in which ophthalmologic consultations were requested by a non-ophthalmologist (OER consultations). The following parameters were compared: total number of cases, age, gender, chief complaint/diagnosis categorized into major entities, and discharge vs. hospitalization. For continuous variables a t-test was used and for categorical variables a chi-squared or Fisher’s exact test was used. A 2-sided p value <0.05 was considered statistically significant.

**Results:**

The total number of patients in the lockdown and non-lockdown groups was 486 and 992, respectively, showing a 51% decrease in visits during lockdown. In the non-lockdown and lockdown groups 56% and 61% of patients were male (p = 0.07), with an average age of 42 (range 0–97, SD 23) and 43 (range 0–90, SD 22) years, respectively (p = 0.44). No statistically significant proportional increase was found for any diagnostic category between the OER visits (p = 0.07) and OER consultation groups (p = 0.77). Nevertheless, analysis revealed a non-significant increase in the proportion of eye trauma from 14.8% to 21.2%, and reduction in eyelid conditions from 10.7% to 5.8%. The total number of OER visits demanding urgent intervention on admission was 43 (non-lockdown) and 24 (lockdown), while hospitalization ratio (hospitalizations/visits) was 8.8% and 10.6%, respectively (p = 0.44).

**Conclusions:**

During the COVID-19 lockdown the guideline for patients in Israel was to avoid unnecessary hospital visits. Since patients tended to avoid the OER rather uniformly regardless of their specific eye condition, determining the risk-benefit of such recommendations and identifying high-risk sub-populations are critical public health issues.

## Introduction

In the beginning of 2020 the first cases of the 2019 coronavirus disease (COVID-19) were detected in Israel. As the incidence of COVID-19 rose [1], the government declared a national state of emergency on March 2020, and a full, general lockdown was announced [2]. The lockdown was accompanied by a recommendation to avoid visiting the emergency room (ER) unless deemed necessary. Worldwide, a few public health studies have assessed the effects of the COVID-19 pandemic on general ER visits, demonstrating a sharp reduction in visits after declaration of a lockdown [3, 4]. This observation raised concerns regarding the risk/benefit of discouraging ER visits during the COVID-19 pandemic, even for severe illness requiring immediate medical intervention [5–7]. While an equivalent reduction in the rate of visits to the ophthalmologic ER (OER) during lockdown is likely [8], the demographics and ocular conditions at presentation may also vary between lockdown and non-lockdown periods. Furthermore, it is possible that government directions will not be similarly accepted and followed in different countries, cultures, age groups, genders, etc., creating differential effects depending on such parameters.

Reasons for OER visits include ophthalmic conditions requiring urgent assessment and intervention such as trauma and loss of vision, as well as a significant proportion of non-urgent conditions such as conjunctivitis [9, 10]. The latter tend to increase as access to ophthalmologic services at primary care settings is reduced, and waiting time increased [11, 12]. Any decrease in OER visits during lockdown may reflect several underlying tendencies: a tendency to avoid presenting with non-urgent conditions as requested by the health authorities; and a general reduction in trauma cases resulting from fewer people leaving their homes during lockdown, and thus fewer work, sport and recreation-related injuries, as has been described [13, 14]. Alternatively, it may reflect avoidance of leaving home and accessing the OER even when sight threatening conditions exist [15]. This might occur either due to fear of contracting the COVID-19 virus, fear of spreading the virus to immediate household family members, and concern over breaking lockdown curfew rules. An additional reason which may be overlooked is that in many ocular emergencies there is no ocular pain, and signs and symptoms may be subtle [16]. This is why a substantial proportion of OER referrals are secondary to examination findings during a routine examination by an eye care practitioner, such as primary ophthalmologists. Such examples include retinal tear, macula-on retinal detachment, and high intraocular pressure. In many countries, optometrists, who serve as eye care practitioners, are another source of referral. During COVID-19, in certain countries optometrists helped coping with the reduced hospital capacity and were responsible for managing the majority of urgent patients [17], while in other countries optometrist services declined drastically [18, 19].

In addition to the health administration recommendation to avoid unnecessary ER visits, many ophthalmological societies recommended avoiding all ocular treatments unless they were urgent [8]. There are four possible reasons for this. First, patients who visit ophthalmic clinics are typically older and thus have a higher risk of poor outcome in the event of a COVID-19 infection. Second, many ophthalmologic procedures are elective. For example, the most common ophthalmic surgery is cataract surgery, which is performed electively [20]. Another factor is that ophthalmologists are at high risk of contracting COVID-19 because of their close contact with patients. Further, the coronavirus is highly prevalent on the ocular surface and nasopharyngeal mucosa, and conjunctivitis can be an early sign of infection [21–23].

The impact of COVID-19 on the ophthalmological patient volume and its potential long-term consequences has been the focus of several studies. The reduction in ophthalmologic procedures, such as vitrectomies for retinal diseases and glaucoma filtering surgeries, may cause disease progression and a guarded prognosis [24]. Studies show that one consequence of the reduction in number of intravitreal injections for macular degeneration during quarantine resulted in considerable vision loss [25]. The decrease in corneal donations owing to the risk of COVID-19 transmission, though very low, has resulted in a major reduction in corneal tissue availability and delays in sight-saving procedures [26]. In youngsters, a delay in diagnosis of amblyopia may result in lifelong visual loss [27]. Thus, determining the risk-benefit of a recommendation to avoid ophthalmologic therapy unless absolutely necessary (which may be difficult for a patient to assess), is a public health issue of paramount importance.

This study quantified and characterized changes in OER encounter patterns during an acute lockdown due to the COVID-19 pandemic in a tertiary public hospital as compared to a parallel period prior to the pandemic. Understanding the influence of government recommendations on patients’ tendency to visit the OER in different countries and centers might aid future public guidance during times of crisis.

## Methods

### Study design

A register-based retrospective cross-sectional study was performed in the Department of Ophthalmology and ER at Rambam Health Care Campus (Rambam), a tertiary level hospital treating approximately 4,000 OER encounters annually.

### Ethics statement

The study received approval from the Rambam Institutional Ethics Committee and was performed in accordance with the declaration of Helsinki. The requirement for informed consent was waived by the ethics committee for all patients, including minors. All patient data were fully anonymized.

### Patients

All records of patients who visited the Rambam OER during the first COVID-19 lockdown (lockdown group), between March 15, 2020 and April 30, 2020 (six-week duration), were retrieved from the hospital’s electronic medical record database. The corresponding time period in 2019 (March 15, 2019 to April 30, 2019) was used as a control (non-lockdown group). For all cases in both groups, the following parameters were extracted: age, gender, and whether they presented with a primary eye condition (OER visit) or a systemic condition for which an ophthalmologic consultation was requested by a non-ophthalmologist physician (OER consultation). Additional extracted parameters for the OER visit group included admittance for in-house hospitalization and a main diagnosis at OER discharge. For OER consultations the consultation query, rather than their systemic diagnosis, was recorded. International Classification of Diseases 10 (ICD-10) diagnoses for OER visits were classified into six major diagnostic categories: eyelid disorders, conjunctival disorders, corneal disorders, retinal disorders, trauma, and others. The “other” diagnostic category included: glaucoma, uveitis, optic nerve disorders, diplopia/strabismus, headache and associated ocular pain, lens disorder, endophthalmitis, unspecified visual disturbance, and a patient encounter for examination and observation without any complaint-related ophthalmological findings. If there was no relevant ICD-10 diagnosis the case was classified as unknown (S1 Table).

In the OER consultations group, the consultation question was classified into five diagnostic categories: fundus, trauma, pain/redness, visual impairment, and other, which contained diagnoses that did not fall under the existing categories. The fundus diagnostic category includes ruling out papilledema or retinal bleeding without the presence of ophthalmic complaints. If no consultation question was recorded the case was classified as unknown (S2 Table). Of note, pediatric patients were included in the study. Although in pediatric ophthalmic clinics the ocular conditions may differ significantly from those of adult patients [28], we found that in the ER the reasons for encountering OER are similar, thus, the same categories were applied for adult and pediatric patients.

### Statistical analyses

After verifying that conditions of validity were met, continuous variables were compared using a t-test. For categorical and binary variables a chi-squared test was used when sample sizes in all categories were adequate, and a Fisher’s exact test used otherwise. The previously described diagnostic entities were treated as a single categorical variable and examined for statistically significant differences in the distribution of categories, and row-wise Fisher’s tests with a Bonferroni correction were applied. A 2-sided p-value <0.05 was considered statistically significant. The statistical analysis was performed using R software, version 4.0.0 (R Core Team, Vienna, Austria).

## Results

The total number of OER encounters during the 6-week COVID-19 lockdown was 486 as compared to 992 in the equivalent 6-week period a year earlier, showing a 51% reduction in visits; 56% vs. 61% of the non-lockdown and lockdown cohorts were male (p = 0.07) with a mean age of 42 (range 0–97, SD 23) and 43 (range 0–90, SD 22) years, respectively (p = 0.44). Demographic characteristics (Table 1) did not differ significantly between the COVID-19 lockdown and non-lockdown groups. The study included patients of all ages to better characterize differences in age-dependent tendencies to avoid OER during acute COVID-19 lockdown.

**Table 1:** Demographics variables of ophthalmologic emergency department encounters in non-lockdown and COVID-19 lockdown groups.

	*Non-lockdown (N = 992)*	*COVID-19 lockdown (N = 486)*	*P value*
***Gender***			0.07
*Female*	437 (44.1%)	190 (39.1%)	
*Male*	555 (55.9%)	296 (60.9%)	
***Age (years)***	42 (SD = 23, R = 0–97)	43 (SD = 22, R = 0–90)	0.44
***Referral***			0.33
*OER consultations*	504 (50.8%)	260 (53.5%)	
*OER visits*	488 (49.2%)	226 (46.5%)	

Abbreviations: OER = Ophthalmologic Emergency Room, SD = Standard deviation, R = Range

Of note, a constant annual elevation in patient numbers was observed in Rambam hospital OER encounters between 2011–2019, with 2341, 2482, 2788, 2979, 3200, 3437, 3757, 3801, and 4013 patients presenting each year, respectively, while in 2020 the number of annual visits dropped to 2993 patients.

**Fig 1:**
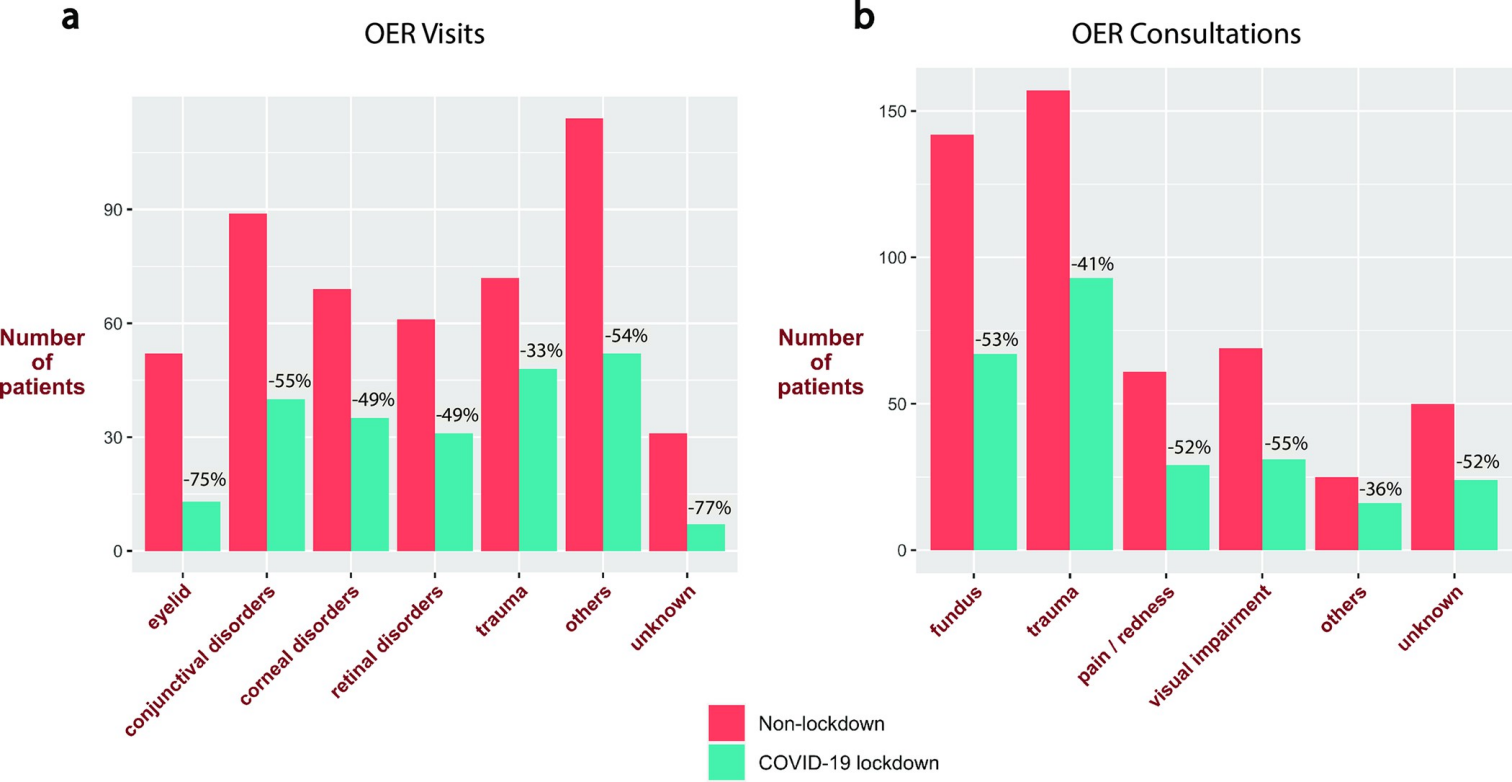
Comparison of non-lockdown and COVID-19 lockdown absolute patient counts by diagnostic categories between (a) OER visits and (b) OER consultations. The change in patient numbers between non-lockdown and COVID-19 lockdown groups is expressed in percent.

**Fig 2:**
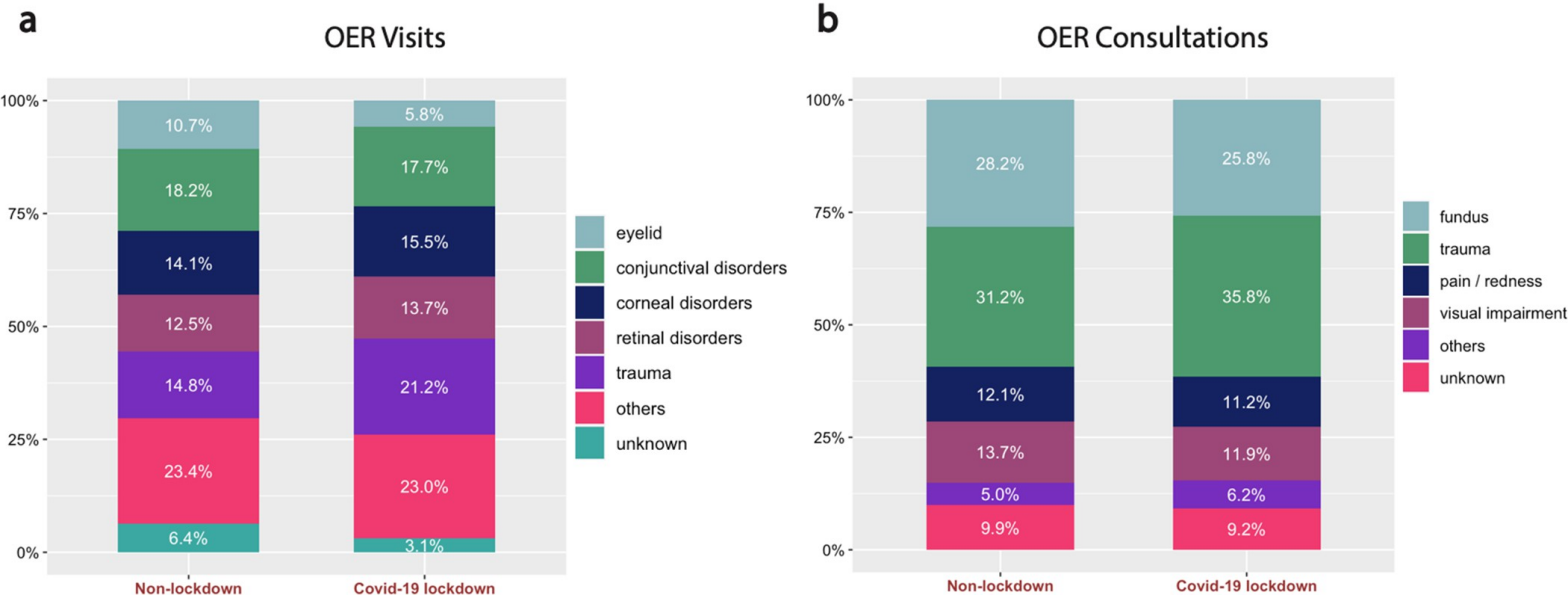
Distribution of diagnostic categories in the non-lockdown and COVID-19 lockdown groups between (a) OER visits and (b) OER consultations. In all graphs the proportion of each diagnostic category from total patients in that group is expressed in percent.

Due to the fact that the COVID-19 lockdown guidelines recommended avoiding unnecessary hospital visits, it is reasonable to assume that the chief complaints in OER visits and consultations would change differentially between the lockdown and non-lockdown periods with relation to characteristics such as severity or pain. We classified OER visits and consultations into diagnostic categories, as detailed in the methods, and evaluated both the difference in absolute numbers and proportions in our diagnostic categories (described above) between the COVID-19 lockdown and non-lockdown groups (Table 2). There was an absolute reduction of 33%-75% in each diagnostic category for OER visits (Fig 1A). Surprisingly, there was no statistically significant change in the distribution of diagnostic categories between lockdown and non-lockdown groups (p = 0.07, Fig 2A). Additionally, a detailed evaluation of entities within the “other” diagnostic category revealed no statistically significant change in the proportion of any entity (Table 3). Similarly, there was a reduction of 36%-55% in each diagnostic category for OER consultations (Fig 1B) without differences in the distribution of diagnostic categories between lockdown and non-lockdown groups (p = 0.77, Fig 2B).

**Table 2:** Distribution of diagnostic categories between OER visits and consultations in the non-lockdown and COVID-19 lockdown groups.

	*Non-lockdown (N = 992)*	*COVID-19 lockdown (N = 486)*	*P value*	*Adjusted P value^#^*
***OER Consultations- Diagnostic categories***		N(%)	0.77*	
*Fundus*	142 (28.2%)	67 (25.8)	0.49^+^	1.00
*Trauma*	157 (31.2%)	93 (35.8)	0.22^+^	1.00
*Pain/ redness*	61 (12.1%)	29 (11.2)	0.72^+^	1.00
*Visual impairment*	69 (13.7%)	31 (11.9)	0.57^+^	1.00
*Others*	25 (5.0%)	16 (6.2)	0.50^+^	1.00
*Unknown*	50 (9.9%)	24 (9.2)	0.79^+^	1.00
***OER Visits- Diagnostic criteria***			0.07	
*Eyelid disorders*	52 (10.7%)	13 (5.8)	0.04^+^	0.25
*Conjunctival disorders*	89 (18.2%)	40 (17.7)	0.92^+^	1.00
*Corneal disorders*	69 (14.1%)	35 (15.5)	0.65^+^	1.00
*Retinal disorders*	61 (12.5%)	31 (13.7)	0.63^+^	1.00
*Trauma*	72 (14.8%)	48 (21.2)	0.04^+^	0.28
*Others*	114 (23.4%)	52 (23.0)	1.00^+^	1.00
*Unknown*	31 (6.4%)	7 (3.1)	0.08^+^	0.53

* Fisher’s exact test on entire contingency table.

^+^ row-wise Fisher’s exact tests.

^#^ Bonferroni correction

Abbreviations: OER = Ophthalmologic Emergency Room.

**Table 3:** Distribution of diagnostic entities included in the “other” diagnostic category between OER visits in the non-lockdown and COVID-19 lockdown groups.

	*Non-lockdown (N = 114) N(%)*	*COVID-19 lockdown (N = 52) N(%)*	*P value*	*Adjusted P value^#^*
**“Others” category entities**			0.40	
*Diplopia/ strabismus*	4 (3.5)	2 (3.8)	1^+^	1
*Encounter for examination*	48 (42.1)	16 (30.8)	0.17^+^	1
*Endophthalmitis*	4 (3.5)	1 (1.9)	1^+^	1
*Glaucoma*	11 (9.6)	8 (15.4)	0.30^+^	1
*Headache and ocular pain*	11 (9.6)	2 (3.8)	0.35^+^	1
*Lens disorder*	3 (2.6)	3 (5.8)	0.38^+^	1
*Optic nerve disorder*	8 (7.0)	2 (3.8)	0.73^+^	1
*Unspecified visual disturbance*	9 (7.9)	8 (15.4)	0.17^+^	1
*Uveitis*	16 (14.0)	10 (19.2)	0.49^+^	1

* Fisher’s exact test on entire contingency table.

^+^ row-wise Fisher’s exact tests.

^#^ Bonferroni correction.

Abbreviations: OER = Ophthalmologic Emergency Room.

Although the overall distribution of diagnostic categories did not differ significantly between the two periods, some trends in entities can be pointed out and are reflected by the uncorrected row-wise p-values. There was a reduction of OER visits due to eyelid abnormalities such as blepharitis and chalazion from 10.7% in the non-lockdown group to 5.8% in the lockdown group, while the proportion of visits due to trauma increased from 14.8% to 21.2%, respectively.

There was a reduction in total numbers of patients requiring urgent intervention who were subsequently admitted, from 43 admissions in the non-lockdown period to 24 admissions in the lockdown group, but the admission rate did not differ significantly and was 8.8% and 10.6%, respectively (p = 0.44).

## Discussion

The aim of this study was to evaluate the effect of the COVID-19 lockdown on the patient population presenting to the OER. The results reveal a significant reduction in patient encounters at Rambam hospital OER during the COVID-19 lockdown, without a statistically significant difference in the distribution of diagnostic categories among OER visits between the lockdown vs. non-lockdown groups.

We expected to observe a reduction in absolute patient counts, but the distribution of diagnostic categories and relative reduction of severe and urgent cases were unknown. It is important to study these parameters in order to assess the short- and long-term effects of an acute lockdown on our ophthalmic patient population, particularly those suffering from urgent ophthalmologic conditions or chronic ophthalmic conditions which may require immediate attention. Ultimately, such data can help finetune the correct instructions that should be given during acute lockdown to best balance the risk of leaving home and attending the ER, against refraining from receiving acute medical care.

With the implementation of the first COVID-19 lockdown, the Israeli ministry of health advised citizens to stay at home, seek medical advice by phone when possible, and unless absolutely necessary avoid presenting to the hospital and scheduling an ophthalmic examination with a primary care ophthalmologist [29]. The purpose of these guidelines was to reduce COVID-19 infection as well as to leave the ER vacant for COVID-19 cases. In a literature review by Uscher-Pines et al. [30], the average fraction of non-urgent ED visits during routine work in the US was 37% while only 63% were classified as visits with conditions for which a delay of several hours would increase the likelihood of an adverse outcome. It is thus reasonable that non-urgent ER visits were reduced to minimize viral exposure and diminish workload in hospitals, thus enabling channeling of resources towards treatment of COVID-19 patients.

The observed uniform decrease in OER visits implies that in this patient cohort the COVID-19 lockdown had an undesired effect in that urgent diagnoses may have been delayed or deferred, rather than a specific reduction in non-urgent OER visits. Interestingly, there was no statistically significant difference between patients’ gender and age in the lockdown vs. non-lockdown groups. While we expected older individuals to avoid the ER far more than younger individuals, this was not evident in the data. Moreover, there was no statistically significant difference within each diagnostic category for OER consultations, reflecting that the pandemic did not change the tendency of doctors in the ER to refer patients to an ophthalmic examination. Strikingly, the distribution of diagnostic categories among OER visits also remained largely unchanged. There may be a rationale behind a decrease in the absolute number of trauma cases requiring OER treatment due to less professional hazards as well as outdoor and sports activities during lockdown. The same cannot be presumed regarding the incidence of conditions such as uveitis, retinal tears, or detachments, which are presumed to remain similar. The sharp decrease of 75% and 55% in patients presenting to the OER with retinal detachment and retinal tears, respectively (S1 Table), may thus reflect undertreatment of serious ophthalmic conditions due to OER avoidance.

Two possible explanations can be proposed: One explanation may be patients’ lack of knowledge regarding sight threatening and ophthalmic emergencies which require urgent treatment. Another contributing factor can be inadequate availability of community ocular services leading to fewer patients being referred to the OER, alongside insufficient knowledge regarding ophthalmic urgent conditions among primary care providers.

This study’s results are similar to findings from worldwide studies; Nair et al. reported that 72.5% of ophthalmologists did not see patients during the pandemic in a survey that included over 1000 ophthalmologists in India [31]. Al-Khersan et al. reported a 90% reduction in ophthalmic surgical procedures during April 2020 as compared to the same month a year earlier at the Bascom Palmer Eye Institute, a large tertiary hospital in Miami [32]. In that study, a younger mean age was observed among both patients and surgeons during the pandemic, reflecting the fact that not only patients but also physicians may be affected by fear of the pandemic. In a recent study, Poyser et al. reported a 53% reduction in OER visits in a tertiary hospital in the UK [33]. In contrast to the results shown here, they reported a shift in the three most common reasons for OER visits with conjunctivitis and blepharitis being replaced by uveitis and keratitis. These differences may be due to factors such as patient education and broader use of telemedicine in the UK [34–36] that may limit unnecessary referrals. Additionally, in the UK, optometrists provide primary ocular care, including treatments for ocular hypertension and glaucoma, low vision, cataract, and red eye, presumably leading to higher availability of primary ophthalmologic in the UK [37].

One step taken to mitigate the impact of ophthalmological societies’ recommendations to avoid all non-urgent treatments is the development of new medical record-based triage systems. The risk-benefit of delaying treatment was evaluated for procedures like intravitreal injections for macular diseases, allowing a better-guided decision with greater patient confidence [16, 38]. Such systems are easier to implement with patients who regularly receive treatment, but they can also be integrated into OER by using telephonic triage and developing referral guidelines for primary care providers. Another consequence of the significant reduction in OER visits is the impact on ophthalmology trainees, who are exposed to fewer patients and ophthalmic emergencies, as well as a gap in didactic teaching. Web-based teaching, virtual surgical simulators, and tele-mentoring can ensure the continuity and effectiveness of ophthalmology training [39].

This study has several limitations. It was conducted in a single tertiary hospital. Integrating information from different centers as well as community health care centers can provide a broader perspective. Additionally, the retrospective nature of this study makes it vulnerable to bias associated with usage of electronic health record data [40]. The decision to use only coded data (without free text) aimed to maximize data accuracy.

Although the study was conducted on data collected during a pandemic, its conclusions may be inferred to other potentially similar emergency settings. For example, in Israel ER visits may decline during times of high tension (e.g., terrorist attacks, riots, war), particularly in targeted areas where people may prefer to stay at home. It is thus crucial to establish an organized, evidence-based approach to emergency recommendations. Such an approach should include reliable data sources for patients and primary healthcare providers, including detailed recommendations tailored to specific ocular conditions spanning chronic diseases and emergencies.

## Conclusions

While OER visits can be effectively minimized during an emergency such as a pandemic, a general recommendation to refrain from unnecessary hospital visits may lead to ophthalmic emergencies left untreated as well as avoidable aggravation of chronic ophthalmic conditions. There is need for an alternative approach integrating resources such as telemedicine and investment in patient education about distinguishing eye symptoms that may reflect serious conditions.

## Supporting information

S1 Table Diagnostic entities, including all diagnostic categories, in OER visits between the non-lockdown and COVID-19 lockdown groups. (TIF)

S2 Table Diagnostic entities, including all diagnostic categories, for OER consultations between the non-lockdown and COVID-19 lockdown groups. (TIF)

S3 Table Non-lockdown OER visits with ICD-10 diagnosis, year of birth and decision regarding hospitalization. (TIF)

S4 Table Lockdown OER visits with ICD-10 diagnosis, year of birth and decision regarding hospitalization. (TIF)
