# Supplementary figures and images for: Ophthalmologic emergency room visits during COVID-19 lockdown–Characterization from Haifa, Israel
Source: PLoS One. 2022 Aug 19;17(8):e0273033. doi: 10.1371/journal.pone.0273033 (PMC9390934; doi:10.1371/journal.pone.0273033)

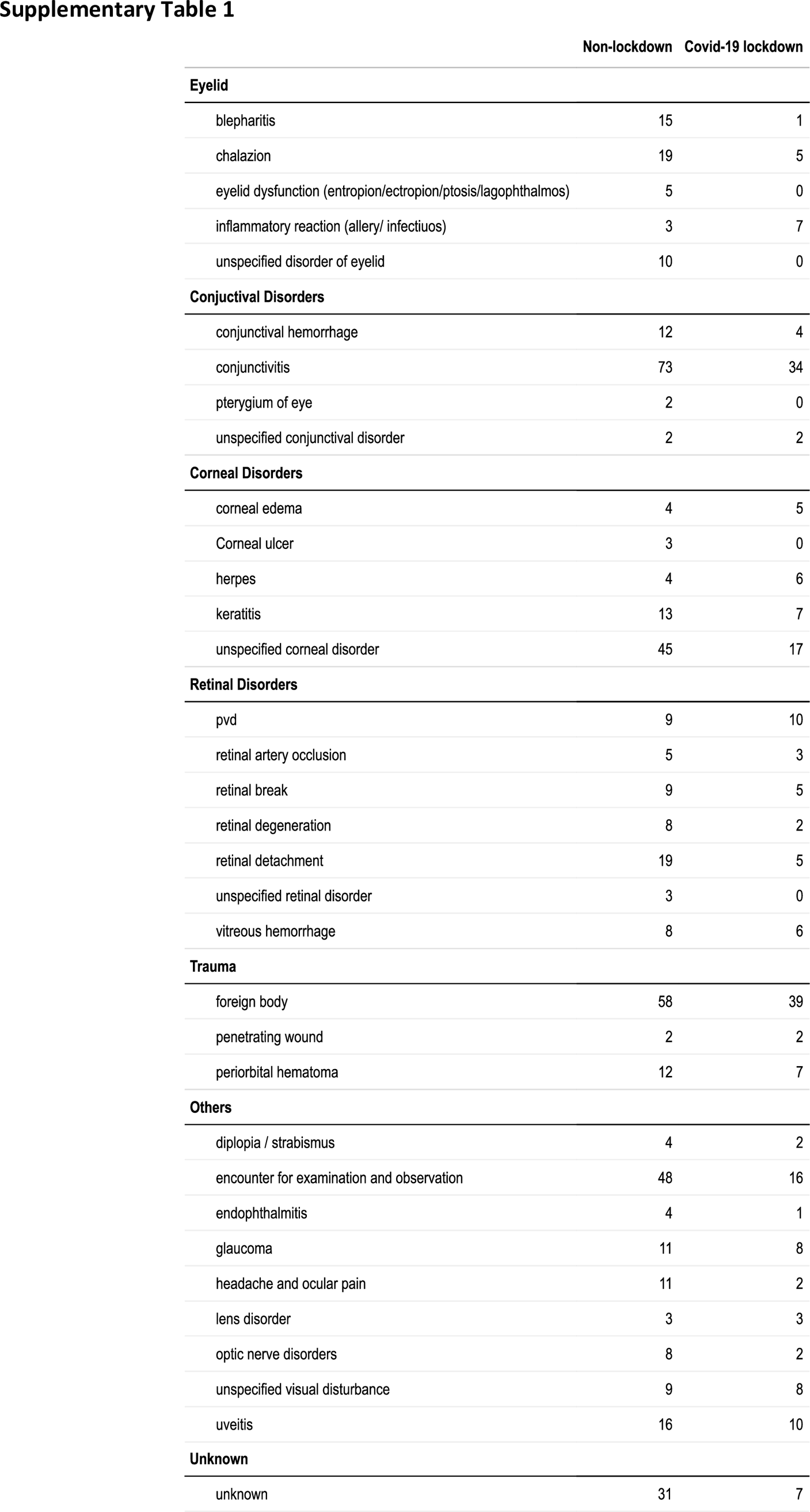

Supplement: S1 Table — (TIF) [file pone.0273033.s001.tif]

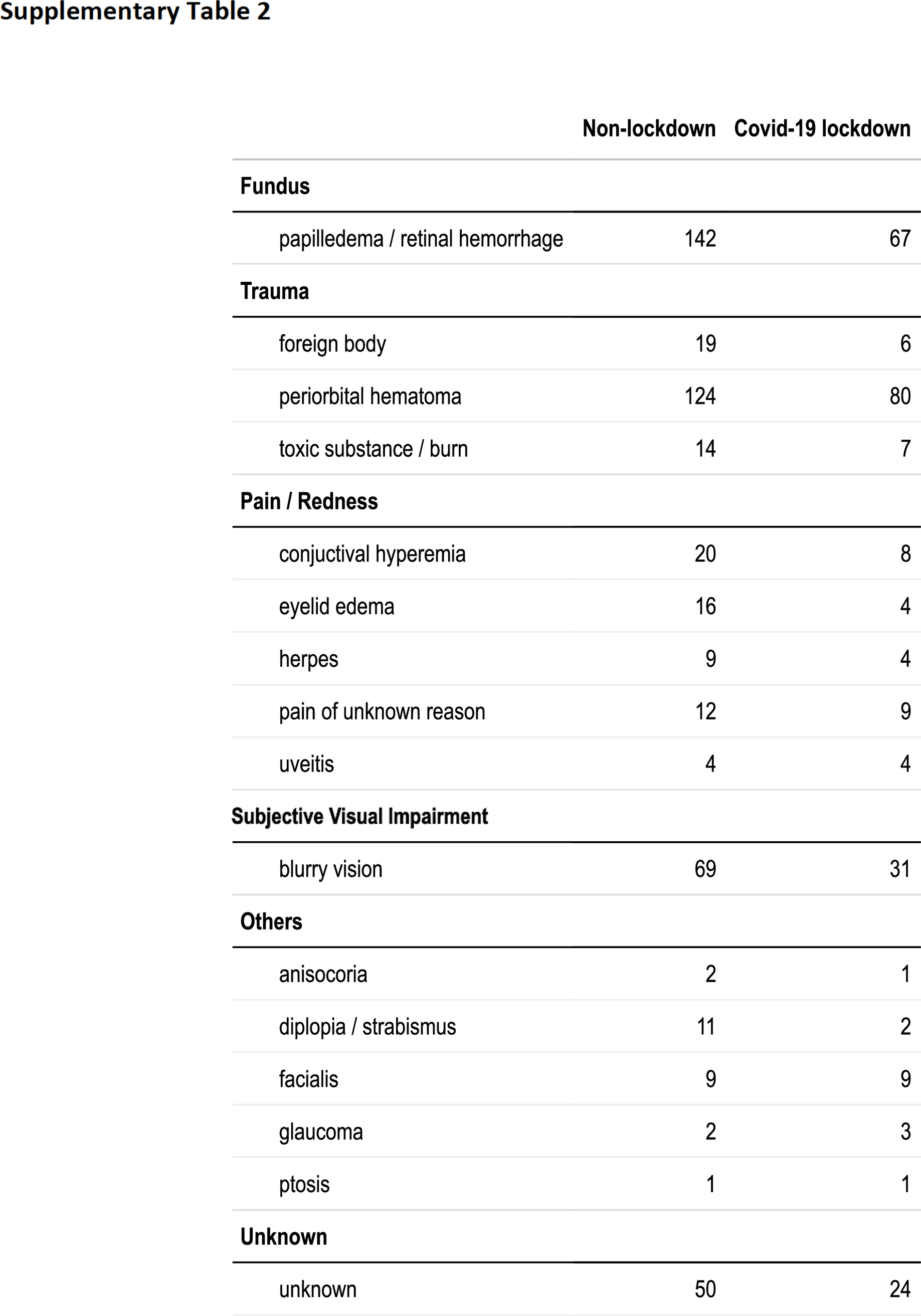

Supplement: S2 Table — (TIF) [file pone.0273033.s002.tif]

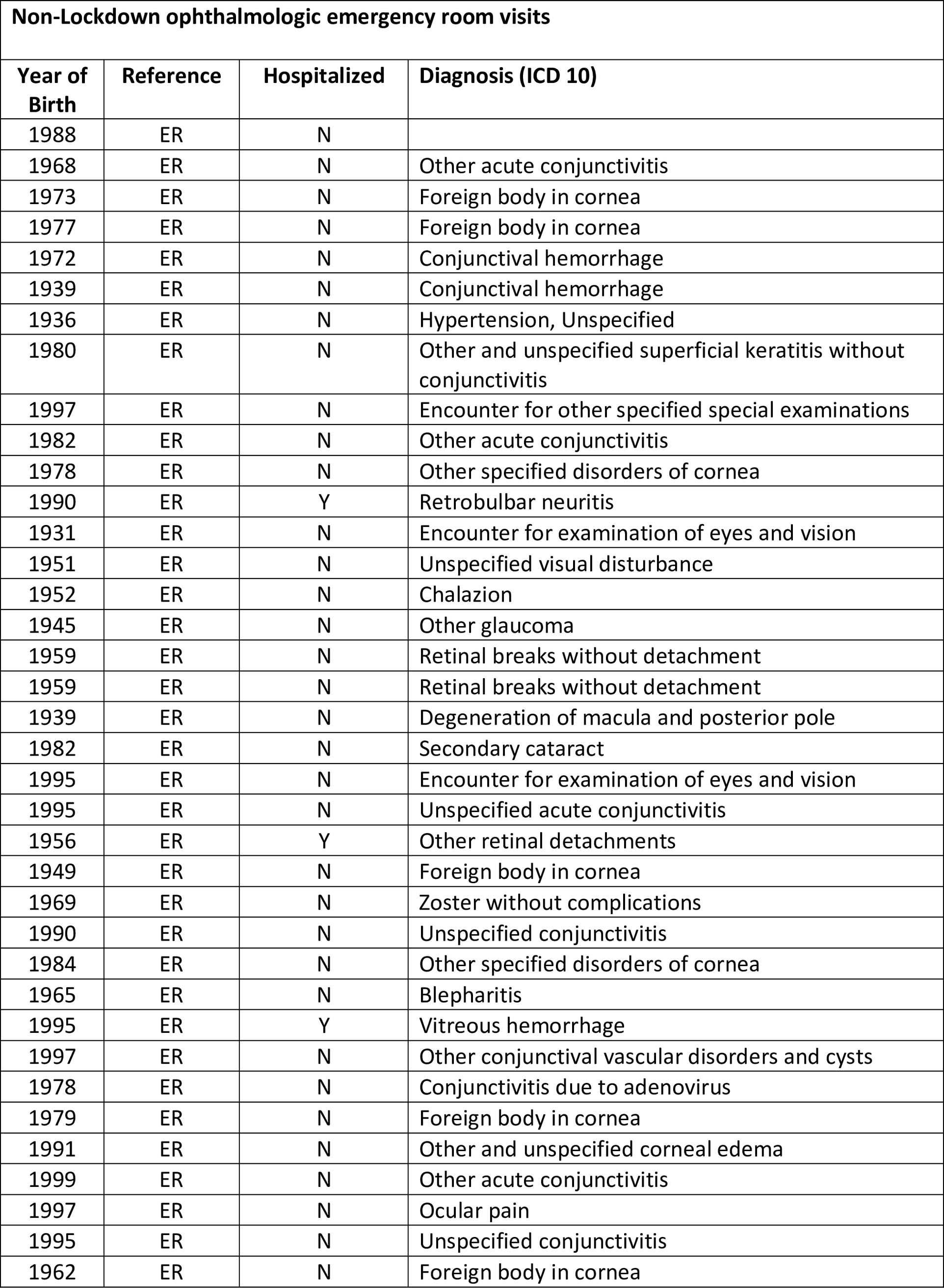

Supplement: S3 Table — (TIF) [file pone.0273033.s003.tif]

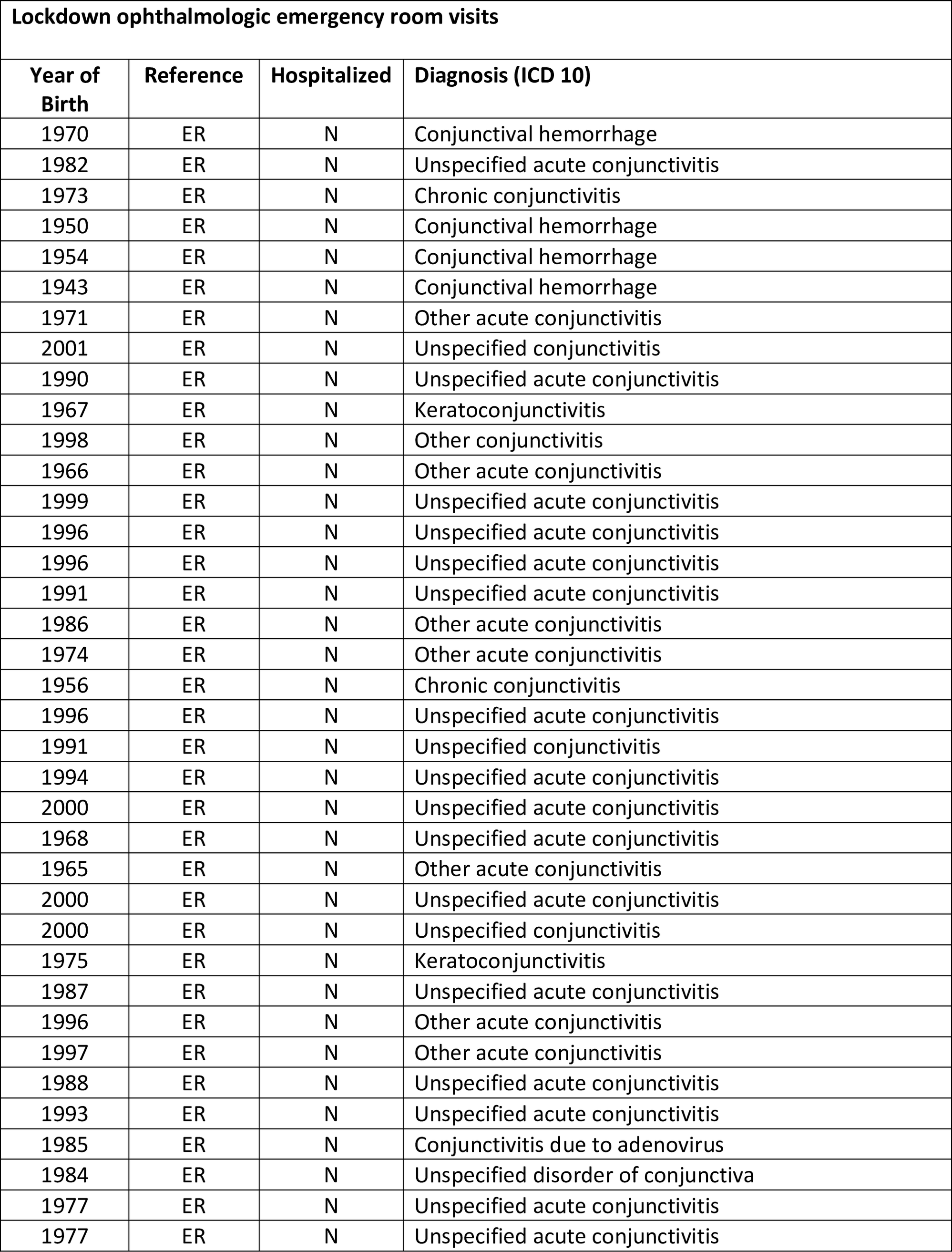

Supplement: S4 Table — (TIF) [file pone.0273033.s004.tif]
